# Structural Integrity of Anterior Ceramic Resin-Bonded Fixed Partial Denture: A Finite Element Analysis Study

**DOI:** 10.3390/jfb14020108

**Published:** 2023-02-15

**Authors:** Mas Linda Mohd Osman, Tong Wah Lim, Hung-Chih Chang, Amir Radzi Ab Ghani, James Kit Hon Tsoi, Siti Mariam Ab Ghani

**Affiliations:** 1 Centre of Restorative Dentistry Studies, Faculty of Dentistry, Universiti Teknologi MARA, Sungai Buloh 47000, Selangor, Malaysia; 2 Division of Restorative Dental Sciences, Faculty of Dentistry, The University of Hong Kong, Hong Kong SAR, China; 3 Department of Biomedical Engineering, Hungkuang University, Taichung City 433304, Taiwan; 4 College of Engineering, Universiti Teknologi MARA, Shah Alam 40450, Selangor, Malaysia; 5 Division of Applied Oral Sciences and Community Dental Care, Faculty of Dentistry, The University of Hong Kong, Hong Kong SAR, China

**Keywords:** finite elemental analysis, lithium disilicate, resin-bonded fixed partial denture, zirconia

## Abstract

This study was conducted as a means to evaluate the stress distribution patterns of anterior ceramic resin-bonded fixed partial dentures derived from different materials and numerous connector designs that had various loading conditions imposed onto them through the utilization of the finite element method. A finite element model was established on the basis of the cone beam computed tomography image of a cantilevered resin-bonded fixed partial denture with a central incisor as an abutment and a lateral incisor as a pontic. Sixteen finite element models representing different conditions were simulated with lithium disilicate and zirconia. Connector height, width, and shape were set as the geometric parameters. Static loads of 100 N, 150 N, and 200 N were applied at 45 degrees to the pontic. The maximum equivalent stress values obtained for all finite element models were compared with the ultimate strengths of their materials. Higher load exhibited greater maximum equivalent stress in both materials, regardless of the connector width and shape. Loadings of 200 N and 150 N that were correspondingly simulated on lithium disilicate prostheses of all shapes and dimensions resulted in connector fractures. On the contrary, loadings of 200 N, 150 N, and 100 N with rectangular-shaped connectors correspondingly simulated on zirconia were able to withstand the loads. However, two of the trapezoidal-shaped zirconia connectors were unable to withstand the loads and resulted in fractures. It can be deduced that material type, shape, and connector dimensions concurrently influenced the integrity of the bridge.

## 1. Introduction

Resin-bonded fixed partial denture (RBFPD) is a minimally invasive treatment option to replace a missing tooth. The main advantages of RBFDP include it simultaneously being a fixed, cost-effective tooth replacement option that readily preserves tooth structure [1]. Despite the cumulative survival rates of an RBFDP being similar to that of a conventional bridgework, less preparation is required of the former [2,3]. RBFDP preparations are conclusively the more conservative option as it only removes up to 14% of tooth structure; the percentage of tooth structure removal is significantly less than a full all ceramic-preparation, which removes up to 71.9% of tooth structure [4]. Dental implant is another alternative tooth replacement option that requires surgical placement; the previously mentioned requirement ultimately deems it a less favorable option for growing and for individuals who have severe co-morbidities. However, implant placement is favorable in terms of its ability to preserve adjacent teeth [5]. One of the main advantages of implant placement is the preservation of adjacent teeth. Comparisons between implant-supported prostheses and RBFPDs show similar longevities in the teeth adjacent to the edentulous space [6]. In terms of overall treatment satisfaction, there were no statistically significant differences between the patients receiving RBFPD and implant [7]. Both treatment modalities are able to replace a single missing tooth space satisfactorily. The overall clinical performance of RBFPDs is comparable to implant-supported crowns and three-unit conventional bridges [8,9].

RBFPD was introduced in 1973 by Rochette as two-retainer prostheses with lingually perforated metal retainers. These perforations allowed macro-mechanical retention and were bonded to abutment teeth using acrylic resin to the acid-etched enamel [10]. Over the years, there has been a transformation in the overall RBFPD retainer design, from perforated to non-perforated, and from fixed–fixed design to single retained cantilever. These changes occur due to significant developments in materials, adhesion, and increased aesthetic demand, all of which inevitability influenced material and design choices. The transition from a two-retainer RBFPD to a one-retainer was due to the fracture or debonding of one of the retainers [11]. The fracture occurred due to dissimilar movement of the two abutment teeth causing loosening of the cement layer or fracture of the prostheses. The shear peel force affecting the two retainers is reduced in a cantilevered RBFPD [12]. As such, a cantilevered RBFPD has higher survival rates in anterior teeth and will be the prostheses tested in this study [9,13].

Computer-aided design (CAD) and computer-aided manufacturing (CAM) in dentistry have increased in popularity over the years. With CAD and CAM, clinicians are able to design, manufacture, and provide prostheses to their patients in a fast and efficient manner. RBFPDs have a higher success rate, yielding optimal results in the anterior teeth compared to posterior, and in the maxillary arch more than the mandibular, thus highlighting its need for good aesthetic appearance [14]. RBFPDs can be made from metal-ceramic or all-ceramic material. The metal-ceramic prostheses will have metal alloy as the framework and ceramic veneering at the pontic. Conversely, the all ceramic prostheses are fabricated from CAD-CAM technology. Silica-based ceramics such as lithium disilicate have excellent optical and aesthetic properties from their glassy content but have a lower flexural strength [15]. Zirconia, a non-silica ceramic, has excellent flexural strength properties; however, it is an opaque material [3]. To reduce its opacity, a layer of porcelain veneer is often used to improve appearances, similar to the veneering of the metal framework in porcelain fused to metal. The layer of veneering made it susceptible to chipping, which resulted in compromised longevity [16,17,18]. The introduction of monolithic highly translucent zirconia omits the use of a veneering ceramic but utilizes the strength of zirconia. This is advantageous anteriorly where the thin metal wing in an anterior metal-ceramic RBFPD can sometimes be seen through the enamel interface but not with the zirconia [19]. Both monolithic zirconia and IPS lithium disilicate are excellent alternatives to metal-ceramic RBFPDs anteriorly as they provide a better aesthetic for comparable material strength. The reported 5-year survival rates for all-ceramic fixed dental prostheses are 87.9% for zirconia and 100% for glass ceramic restorations [19]. 

Clinical and in vitro investigations have reported that the prostheses fracture commonly occurred at the connector region. It was deduced that the size, shape, length, or the connectors as well as their placements collectively influenced the fracture resistance of FDPs [9]. Many of these findings on RBFPDs were based on clinical studies with minimal to no assessment of the resin-bonded design parameters, especially the connector shape, width, and height [20]. In vitro studies are important in forming the basis of treatment before proceeding to clinical studies. However, our search of the literature [21] concluded that the number of studies centered on finite elemental analysis and in vitro studies on all ceramic cantilevered RBFDPs specifically focused on its parameters and connector design were evidently limited. This study was chosen to examine the limitation of the material at the connector region in teeth with reduced clinical crown height. Two ceramic materials (lithium disilicate and monolithic zirconia without veneering) were chosen for their aesthetic properties. 

This study was conducted to evaluate and establish the stress distribution patterns derived from anterior ceramic RBFDPs sourced from different materials (monolithic zirconia and lithium disilicate) and different connector designs (rectangular-shaped and trapezoidal-shaped) using finite element analysis (FEA). 

## 2. Materials and Methods

### 2.1. Set Up of Master Model

The study protocol was approved by the ethics committee of Universiti Teknologi MARA, Malaysia. Two extracted human teeth (1 central incisor and 1 canine) were obtained from Universiti Teknologi MARA, Malaysia, and stored in room temperature sodium chloride 0.9% (RinsCap NS, ANS Medicare, Kota Bharu, Malaysia). A trial setup was conducted by arranging the extracted central incisor and canine tooth in modeling wax (Metrowax, Metrodent Limited, Huddersfield, UK) with a 7 mm gap to stimulate the missing lateral incisor. A 1:1 base and catalyst silicone putty impression (Elite HD+, Zhermack, Badia Polesine RO, Italy) was mixed, and an impression was taken from the buccal surface to be used as an index for tooth setup [20]. The teeth were removed from the modelling wax and putty index. A 0.1 mm even layer of modelling wax coating was applied onto the root surface of the teeth to mimic the periodontal ligaments (PDL). Excess at the root tip was removed to create an even-thickness coat of wax [22].

**Figure 1:**
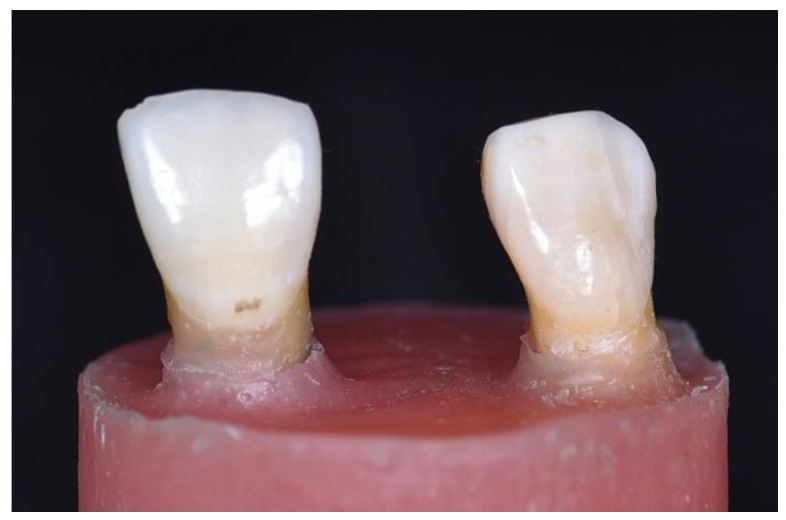
Tooth setup.

For the master model setup, the roots of the extracted teeth were mounted in a cylindrical mold with self-curing acrylic (BasiQ20, Vertex Dental, Zeist, The Netherlands). Teeth were held in place with the putty index during setting to maintain the stimulated spacing. The complete setup is shown in Figure 1. Upon setting, the master model was returned to room temperature sodium chloride 0.9% to maintain teeth hydration prior to preparation. 

### 2.2. Preparation of Abutment and Prostheses

**Figure 2:**
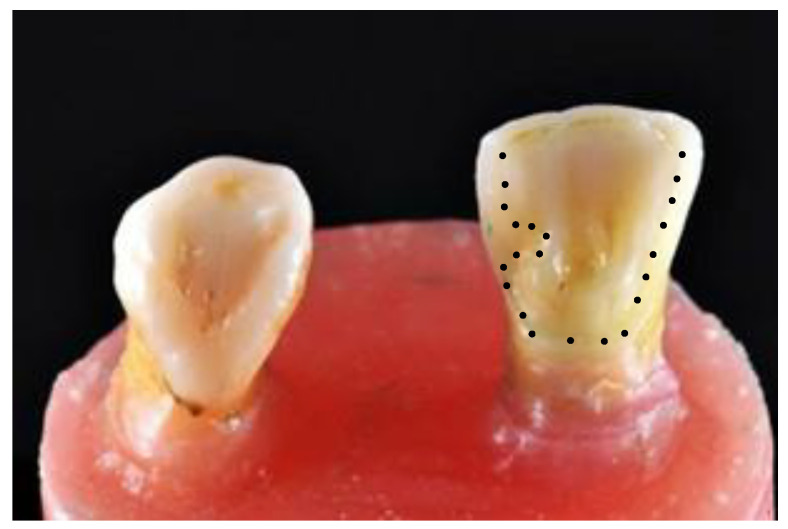
Tooth preparation (margins in dotted lines).

**Figure 3:**
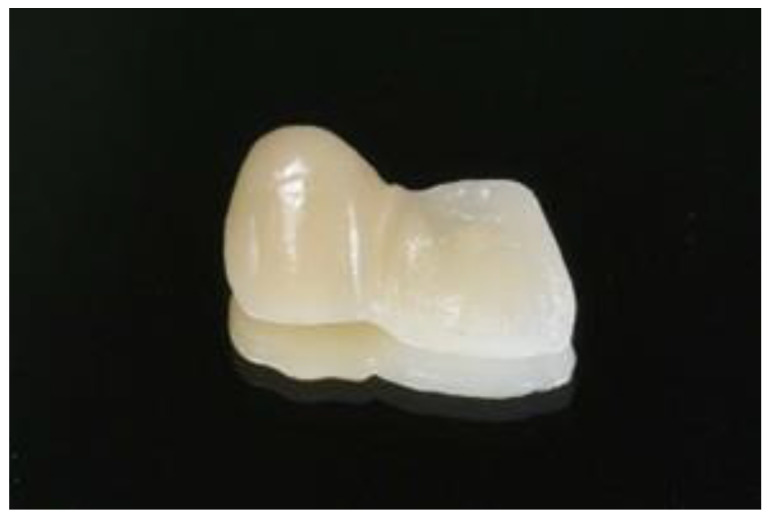
Prostheses.

**Figure 4:**
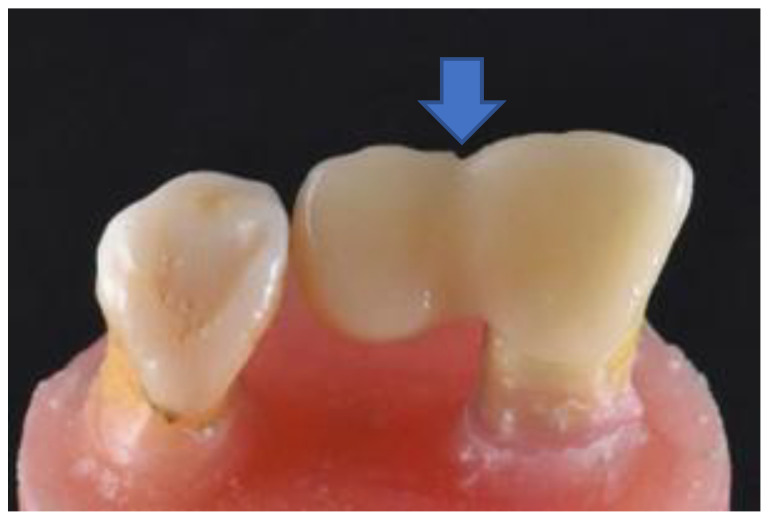
Combined master model that consists of the retainer on the central incisor and the pontic tooth (lateral incisor). The connector is marked by an arrow.

The central incisor was chosen as the abutment. The guidelines for preparation design were to maximize the amount of bondable surface area by lowering the survey line [23,24,25]. Tooth preparation was performed using diamond burs (Shofu, Kyoto, Japan). Preparation of the palatal surface of the central incisor included a fine cervical chamfer (ISO# 101, SF 101), a proximal box on the distal measuring 1.5 mm × 1.0 mm × 0.5 mm, and a pinhole on the cingulum with a ½ round bur (0.5 mm in depth, 1.0 mm in diameter) (ISO# 440). The slight removal of the enamel prisms on the palatal was performed using a football-shaped diamond bur (ISO# 145). All sharp edges and surfaces were smoothened using a white round stone (Figure 2). The tooth preparation was scanned using an intraoral scanner (BenQ Bis 1, AB DentCare Corp., Taipei, Taiwan) to create an STL file. Using three-dimensional (3D) computer-aided design (CAD) software (Exocad GmbH, Darmstadt, Germany), the prostheses were designed with a connector of 5 mm height, 4 mm width, and a retainer thickness of 0.5 mm covering the palatal surface up to the incisal edge. These are the maximum values of our tested prostheses. Once the design had been finalized, the prostheses were milled by a milling machine (imes.350i.core, imes-icore GmbH, Eiterfeld, Hessen, Germany), and Figure 3 shows the completed design. The prostheses and setup were ready for radiograph taking (Figure 4).

### 2.3. Radiographic Imaging of the Master Model

**Figure 5:**
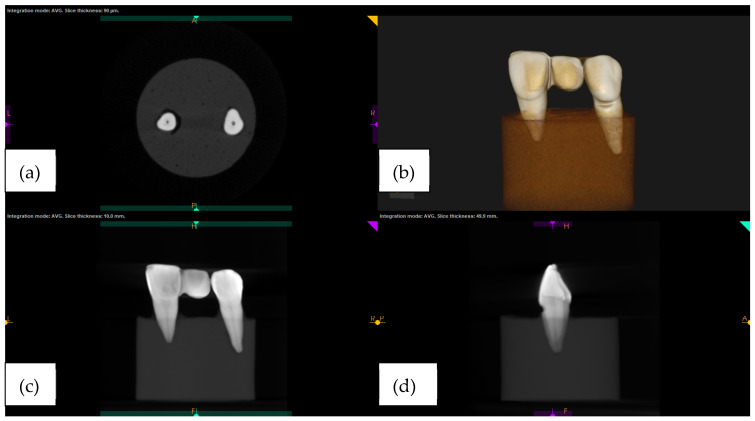
Cone beam computed tomography (CS 3D imaging) of (**a**) axial view; (**b**) 3D image; (**c**) coronal view; (**d**) sagittal view.

A thin layer of petroleum jelly was deliberately applied onto the prostheses fitting surface to preserve space for the cement layer and to maintain its position on the model during imaging. The radiographic image was taken by a cone beam tomography machine (Carestream CS9300, Rochester, NY, USA) with scanning parameters of voxel size 76 µm × 76 µm × 76 µm; tube voltage 64 KV; tube current 5 mA. The radiographic images are as shown in Figure 5. 

### 2.4. Finite Element Modelling and Simulation

**Figure 6:**
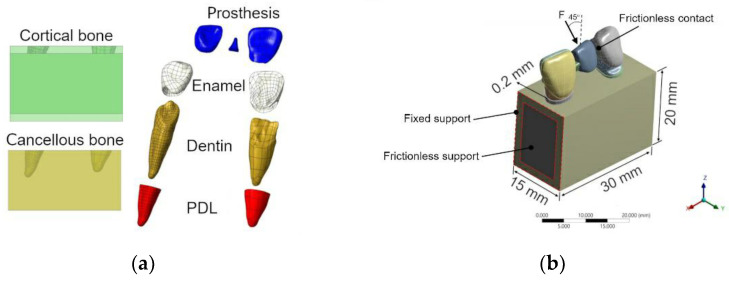
(**a**) Imaged layers of the model. (**b**) The configuration and the boundary conditions of the FE model.

A 3D CAD model was reconstructed using the reverse engineering method. All computed tomography (CT) images were imported to establish 3D models by the image software AVIZO (Thermo Fisher Scientific, Waltham, MA, USA). The CAD software SolidWorks (Dassault Systèmes, Vélizy-Villacoublay, France) was used to assemble the RBFPDs model with different shapes of the connector. Moreover, the bone block and periodontal ligaments (PDL) were also virtually generated by SolidWorks. The thickness of PDL was assumed to be 0.2 mm according to the tooth root surface. The dimensions of the bone block were 30 mm in length, 20 mm in height, and 15 mm in depth, as shown in Figure 6. In designing the bone block, it was assumed that there was no marginal bone loss and that teeth were embedded within the bony complex [26]. All assembled CAD models were exported to ANSYS Workbench 2022 R1 (Ansys Inc., Canonsburg, PA, USA).

The finite element program ANSYS was used to conduct the components that included the mesh generation and the computation of all FE models, comprising the pontic, connector, retainer, enamel, dentin, PDL, and bone block. No cement layer was added in the stimulation as all of the connecting surfaces were assumed to be bonded in the models, except for the surface between the pontic and the canine, which was anticipated to be frictionless. The assembly of finite element models is shown in Figure 6a. An element convergence test was carried out to determine the suitable element size. A solid tetrahedron element was used to mesh the models, and an approximate total of 1,138,226 nodes and 820,127 elements were created. All the materials were assumed to be homogenous, linear elastic, and isotropic. The properties and element size of part are listed in Table 1. These material properties were determined from the literature [27,28,29,30,31]. The materials planned for testing were lithium disilicate (IPS e. max CAD) and 5-yttria/3-yttria stabilized tetragonal zirconia (IPS e. max ZirCAD Prime). 

**Figure 7:**
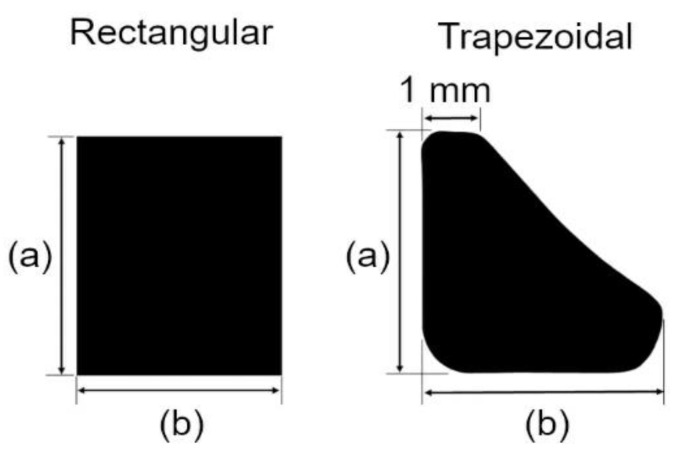
Rectangular and trapezoidal-shaped connector cross-section.

The area between the retainer on the central incisor and the pontic is called the connector. The simulated master model was designed with two cross-sectional connector shapes: rectangular or trapezoidal, as shown in Figure 7. Four models sourced from lithium disilicate and zirconia were simulated into four different models with varied dimensions. In the trapezoidal cross-sectional shape, the 1 mm represented the fixed width with the highest point of the trapezoidal shape. The height (a) and width (b) were the variables in the dimension for tooth shapes. Sixteen models were created, and the dimension parameters are listed in Table 2.

Three loadings of 200 N, 150 N, and 100 N were applied to the palatal surface in the center of the models at 45°. Loading of 200 N was based on previous studies, and the 100 N was based on the reported maximum masticatory bite force [32,33]. The noted clinical differences in terms of masticatory force in different genders and different loading conditions enabled further examination of the potential discrepancies. The edges of the cortical shell at two cut-section faces were fixed. The frictionless support was further applied to the cut section of the bone structure to compensate for the missing bone structure. Frictionless contact was appropriately applied to the surface between the pontic and the supporting tooth as a means of preventing the occurrence of surface penetration. The loading and boundary conditions are shown in Figure 6b. 

## 3. Results

### 3.1. Volume of Tested Connector

The thickness of the connector varied according to the connector’s two-end surfaces joining the area between the retainer and pontic. At the outline of the proximal area between the pontic and abutment tooth crown, which was shaped as an anatomical curve, alterations to the mesio-distal width were made in a incisal-gingival direction. The final volume of the connector from the different dimensions was calculated with ANSYS (Table 3). 

As shown in Table 3, the volume of the connector differed according to its dimensions. The largest connector volume was in the 5 mm × 3 mm rectangular group, followed by 4 mm × 3 mm in the rectangular group and 5 mm × 4 mm × 1 mm in the trapezoidal group. The trapezoidal-shaped connector in the 4 mm × 2 mm × 1 mm group had the smallest connector followed by the 5 mm × 2 mm × 1 mm in the trapezoidal group and 4 mm× 2 mm in the rectangular group.

### 3.2. Intracomparison of the Maximum Equivalent Stress (MES) between Trapezoidal and Rectangular Shape within the Same Material

**Figure 8:**
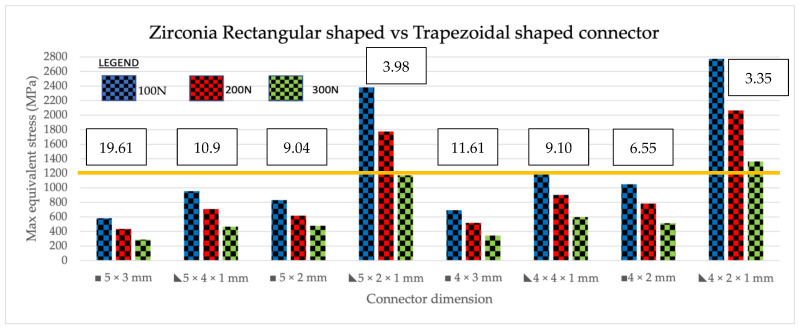
Intracomparison of rectangular vs. trapezoidal connector response from zirconia subjected to 200 N, 150 N, and 100 N load. The orange line indicates the level of flexural strength of zirconia at 1200 MPa. The area above the line indicates the maximum equivalent stress (MES) above 1200 MPa and the material will not survive. The connector volume is stated above the graph in mm^3^.

**Figure 9:**
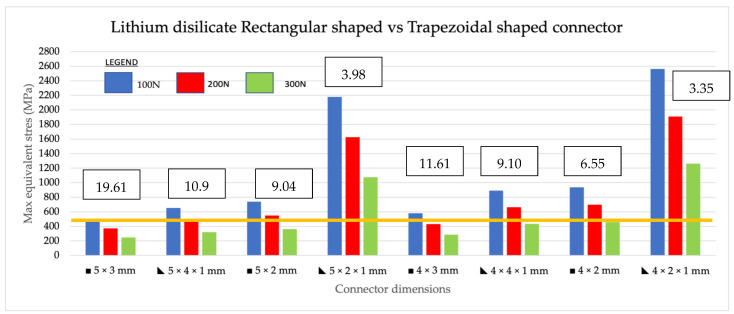
Intra-comparison of rectangular vs. trapezoidal connector response made from lithium disilicate subjected to 200 N, 150 N, and 100 N load. The orange line indicates the level of flexural strength of lithium disilicate at 460 MPa. The area above the line indicates the maximum equivalent stress (MES) above 460 MPa and the material will not survive. The connector volume is stated above the graph in mm^3^.

Maximum equivalent stress, also known as Von Mises stress, is a widely used failure theory that relates to the uniaxial mechanical properties of a multi-axial stress state of a structure when subjected to loading. It is used as a scalar indicator to determine material failure. In the zirconia group, the maximum equivalent stress of the models increased as loading was increased from 100 N to 200 N, for both shapes. In comparing rectangular-shaped connectors with trapezoidal shaped in zirconia (Figure 8), the highest maximum stress values were seen in the trapezoidal-shaped 4 mm × 2 mm × 1 mm group, which had the smallest connector volume of 3.35 mm^3^ under all loading conditions (200 N, 150 N, and 100 N). The second highest maximum stress value was recorded in the 5 mm × 2 mm × 1 mm trapezoidal group, which had the second lowest connector volume of 3.98 mm. These two dimensions had MES exceeding the flexural strength of zirconia material of 1200 MPa in all three loadings. For the lithium disilicate group (Figure 9), similar results were found, albeit with lower maximum equivalent stress values compared to zirconia. Similar to zirconia, the highest stress values were found in the trapezoidal 4 mm × 2 mm × 1 mm group, which had the smallest connector volume followed by the trapezoidal 5 mm × 2 mm × 1 mm. Upon loading of 200 to 150 N, all the connectors exhibited MES higher than the material’s flexural strength of 460 MPa. With increasing loading values, the amount of stress in all dimensions increased relative to the volume or shape of the connector. In conclusion, in zirconia, the two smallest connector volumes exhibited the highest MES and vice versa for the two largest connector volumes, which exhibited the lowest MES in the analysis. In lithium disilicate, the three smallest connector volumes had the highest MES with the three largest volumes exhibiting the three least MES at the connectors.

### 3.3. Intercomparison of Maximum Equivalent Stress between Different Materials with the Same Shape

**Figure 10:**
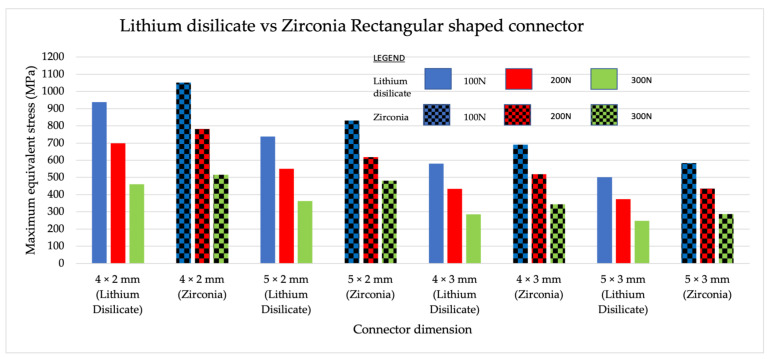
Intercomparison between the rectangular-shaped connectors made from lithium disilicate and zirconia to subjected to 200 N, 150 N, and 100 N load.

**Figure 11:**
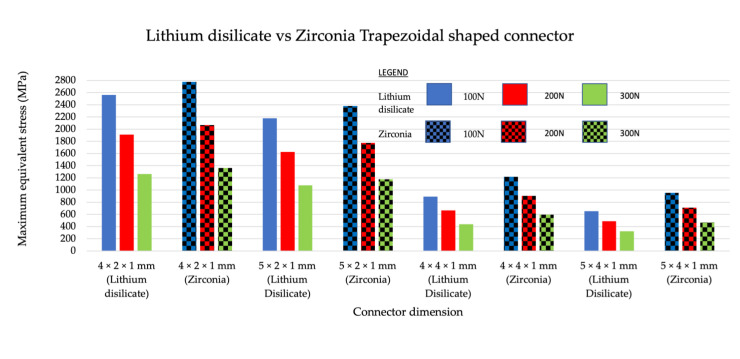
Intercomparison between the trapezoidal-shaped connectors made from lithium disilicate and zirconia subjected to 200 N, 150 N, and 100 N load.

A general comparison made between zirconia and lithium disilicate concluded that zirconia had a higher maximum equivalent stress value in comparison to lithium disilicate when tested with varying loading values of similar dimensions. This was because the zirconia had a higher young modulus value compared to lithium disilicate. Stress is a product of Young’s modulus and strain; therefore, stress was higher in zirconia. When rectangular-shaped connectors in zirconia and lithium disilicate were compared (Figure 10), higher maximum equivalent stress values were recorded in both zirconia and lithium disilicate for the 4 mm × 2 mm group, followed by the 5 mm × 2 mm group. In trapezoidal-shaped connectors, zirconia recorded higher equivalent stress values compared to lithium disilicate for all loading values and dimensions (Figure 11). The 4 mm × 2 mm × 1 mm group made from lithium disilicate recorded higher maximum equivalent stress values. This was followed by a 5 mm × 2 mm × 1 mm group made from zirconia.

### 3.4. Patterns of Stress

**Figure 12:**
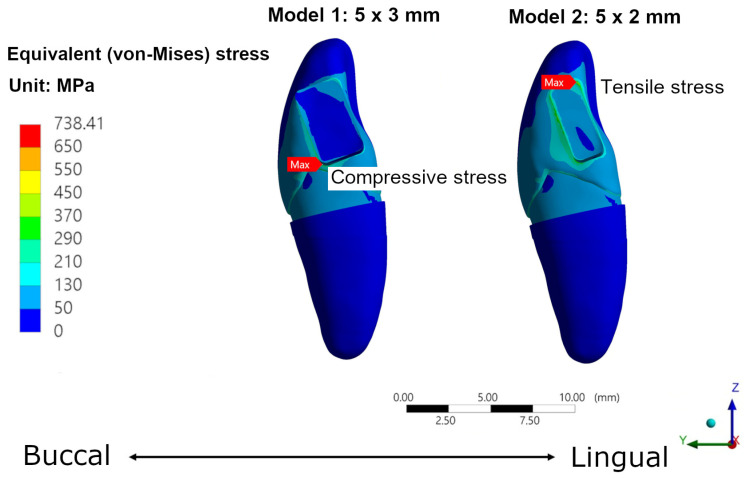
Stress distribution for the FE model.

**Figure 13:**
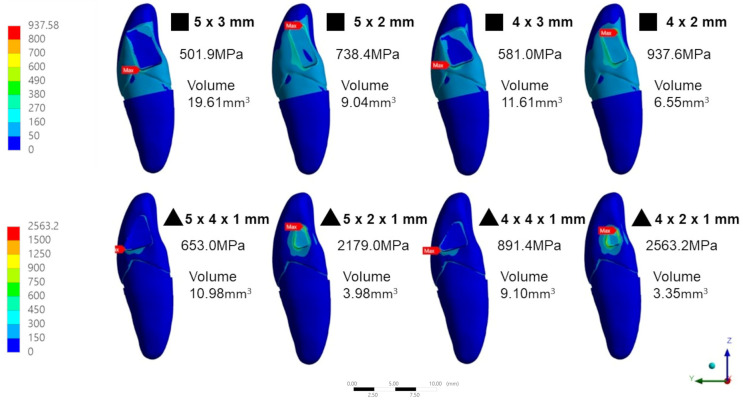
Stress distribution of the FE models of different connector shapes and dimensions.

The stress pattern obtained from different models varied despite all models sharing the same location of the maximum stresses, as well as the edge between the crown and the connector. For both connector shapes, the maximum stress occurred at the connector edge close to the lingual side (tension side) when the base (b) of the connector was 2 mm; the maximum stress occurred at the connector edge close to the buccal side (compression side) when the base (b) of the connector was greater than 2 mm. Two models represented different stress patterns and locations of the maximum stresses (Figure 12). The location of the stress pattern in the eight different connector design and dimensions is shown in Figure 13. The area of maximum stress changed according to the dimensions of the connector area. 

## 4. Discussion

This study used FEM to determine the stress distribution in connectors with different dimensions and cross-sectional shapes made from lithium disilicate and zirconia.

The investigated connector cross-sectional shapes were trapezoidal and rectangular. The following shapes were chosen as the trapezoidal was the anatomical cross-sectional appearance of a central incisor where the cervical portion was wide and became narrower towards the incisal edges. Conversely, a rectangular-shaped cross-section was chosen as it provided good volume. Differences in height and width of the connectors occurred due to limitations in clinical crown height. The height of the connector relied on the height of the available clinical crown height. In a normal and healthy dentition, the height of the clinical crown can be influenced by its wear status, as well as differences in gender and ethnicity. According to the literature, the median length of a central incisor crown in unworn and worn teeth is 11.67 mm (ranges 10.70–13.51 mm) and 10.67 mm (ranges 8.56–13.42 mm), respectively, and according to gender, in males, 10.19 mm, and females, 9.39 mm [34,35]. These are values for a Caucasian patient. In Asian dentition, these values are smaller at 9.98 ± 1.05 mm (males) and 9.45 ± 1.15 mm (females) [36]. These values show the variability in possible connector heights and dimensions of the RBFPD. The disadvantage of a rectangular-shaped connector is that clinically, in a tooth with a taller clinical crown height, the width of the connector dimension may be larger than the bucco-palatal contour of the abutment tooth. This will encroach the palatal contours of the abutment tooth and create a bulky prosthesis on the palatal or lingual surface. In the trapezoidal connector design, as its cross-section will mimic the contours of the tooth, this problem can be avoided. 

All ceramic prostheses that readily features translucency prevailed over the porcelain alternative, due to the former being more aesthetically pleasing than the latter [37]. The translucency parameter of lithium disilicate was similar to enamel and dentine, giving it its life-like value [37]. Lithium disilicate, which is synonymous with glass ceramic, distinctly possesses a higher level of aesthetics in comparison to a 3 yttria zirconia. The introduction of translucent monolithic zirconia aims to reduce opacity and increase its aesthetic quality while eradicating the need for veneering. The translucency of zirconia is made by altering the grain size or by adding the concentration of the yttria [38]. Highly translucent zirconia (5Y TZP), even when compared against lithium disilicate, is still less translucent, but its flexural strength is higher than that of lithium disilicate [39]. One of the limitations of lithium disilicate is its flexural strength. As the translucency of e.max CAD increases, the flexural strength reduces [37,40]. The limiting factor for use of lithium disilicate anteriorly lies in its reduced fracture resistance in comparison to the zirconia [41]. This is supported by our findings, where connectors made from zirconia were able to withstand higher loadings as compared to lithium disilicate.

The connector is the weakest point of a prosthesis. The design of the connector influences the strength of the prostheses [42]. Several in vitro and in vivo studies involving all-ceramic conventional bridges concluded that fractures were associated with geometrical variables including size, shape, and placement of the connectors [43]. Complete failures resulting from fractured prostheses inevitably warrant the need for a remake. This commonly occurred in the anterior regions compared to the posteriors [44]. Clinically, situations involving limited availability of crown height in relation to patients who possessed short worn teeth or misshapen teeth, or patients with micro dentition, require simultaneous acknowledgement and comprehension as the stress-related affects are imperatively significant. Finite elemental analysis (FEA) allows for the examination of stress and strain at multiple points. From the study’s findings, the area of the maximum stress was located at the edge of the crown and connector. This is supported by similar studies on cantilevered bridges as it places stress on the area linking the pontic and the retainer, creating tensile stress on the bonded retainer [45]. In the literature search, the connector heights of 3 mm × 2 mm were mentioned for anterior zirconia all-ceramic RBFPDs [46,47,48,49]. To our knowledge, aside from the three, most clinical studies did not mention the connector values. In this study, a comparison between lithium disilicate and zirconia was made. Despite having the same connector dimensions and design, its response to loading in our finite element study differed. According to our findings, a connector volume at 9.04 mm^3^ in resin-bonded fixed dental prosthesis made from lithium disilicate will survive. This is supported by the manufacturer recommendations in a glass ceramic (IPS e.max press) connector for a cantilevered RBFPD, which was 16 mm^2^. This value, however, did not state if there was a difference in the connector value between the anterior and posterior. The height, however, was mentioned in a study of a maxillary premolar, whereby a connector height of 4 mm was deemed sufficient for an e.max press [50]. Measurements of width were not mentioned. In comparison with our study, both height and width were taken into consideration in an IPS e.max CAD. 

Within the same material, from our results, there were differences in the amount of maximum loading with different connector heights and widths. According to the technical information given by manufacturers for lithium disilicate IPS e.max, the height of the prostheses is more important than its width. This is contradicted by our findings. Even as the height is maintained in both rectangular- and trapezoidal-shaped connectors, alterations to its width caused higher maximum equivalent stress values in both lithium disilicate and zirconia. The results deduced zirconia as the material of choice for patients suffering from reduced clinical crown height; its ability to resist higher loading forces under the circumstances of a constant connector width deems it as the most appropriate option. 

Comparing lithium disilicate and zirconia connectors, its reaction to different loadings differed. The reported flexural strength value from the manufacturer of 3 M Lava Plus for the zirconia was 1200 MPa. With this value, most zirconia prostheses will survive an anterior bite force of 100 N. As shown in Figure 9, most models can withstand the given loads of 100 N, 150 N, and 200 N, except for trapezoidal shapes with 5 mm × 2 mm × 1 mm and 4 mm × 2 mm × 1 mm dimensions. These groups had the smallest connector volumes at 3.98 mm^3^ and 3.35 mm^3^, respectively, emphasizing the effect of connector volume influence on the survival of the prostheses. Different patterns can be seen in lithium disilicate connectors where the load had a significant effect on the stress distribution in the model. Lithium disilicate had a flexural strength of 460 MPa and at a loading force of 100 N; all prostheses survived, except for the trapezoidal shapes with 5.0 mm × 2.0 mm × 1.0 mm and 4.0 mm × 2.0 mm × 1.0 mm dimensions. These groups, similar to zirconia, also had the smallest connector volumes. This reinforces the effect of connector volume on the fracture resistance of the prostheses.

The outcomes of this study indicated that the connector volume is a dominant factor that affects the maximum principal stress of the RBFPDs. It is because the small connector has a small second-moment inertia. According to the previous literature [40], a large second moment of inertia can increase the ability to resist the bending force (cantilever deformation). Moreover, different stress patterns were observed when the size of the connector base varied. Tensile stress may occur at the connector edge of the lingual side when a small connector base (2 mm) was used. It is because the oblique biting force can be divided into horizontal and vertical forces. The bending deformation caused by the horizontal force increased as the size of the connector base decreased. This caused an increase in the tensile stress on the lingual side. In contrast, the larger connector base provided good resistance to bending deformation. The connector will undergo rigid body rotation due to loading, which can relieve high stress from the lingual side and shift to the buccal side to reduce the failure or crack generation of the prostheses. Since zirconia and lithium disilicate are brittle materials, high tensile stress can cause catastrophic failure of the prostheses.

Limitations to the FEA study are owed to the material’s trait of being linear and homogenous; difficulties involving incorporation of bonding effects to the overall results further contributes to this limitation. The outcome of this in vitro study is deliberately centered on testing the smallest connector dimensions of the strongest cantilevered RBFDPs as means of confirming its fracture resistance. It is hoped that a better understanding of the structural integrity in relation to connector design of both connector design of both zirconia and lithium disilicate RBFDPs can be correlated to its clinical success. 

## 5. Conclusions

On the basis of the findings from this study, the following can be concluded:

1. The base of the connector serves as a vital variable that significantly influences fracture strength of the materials tested. Regardless of the connector shape, the base of the connector must be of a sufficient size to be able to resist crack propagation and failure of the prostheses. A small sized connector base may induce more tensile stress concentration on the lingual side compared to a larger sized connector base.
2. When the height of the connector was altered and the width was fixed, there was a higher amount of stress on the prostheses. A higher degree of stress was imposed on the prostheses, as a result of fixed connector width and altered connector height.

## Figures and Tables

**Table 1:** Element size (mm), Young’s modulus values, and Poisson ratio for all components. The compression and tensile strength of both zirconia and lithium is stated for comparison.

Material	Element Size (mm)	Young’s Modulus (MPa)	Poisson Ratio (υ)	Compression (MPa)	Tensile Strength (MPa)	References
Enamel	0.4	95,000	0.3	-	-	[27]
Dentine	0.4	18,600	0.31	-	-	[27,28]
5/3-Yttria stabilized Tetragonal zirconia	0.2	205,000	0.24	192–316	27–70	[29]
Lithium disilicate	0.2	83,500	0.21	1378	82	[27]
Periodontal ligament	0.2	68.9	0.45			[30]
Cortical bone	0.8	13,700	0.3	-	-	[31]
Cancellous bone	0.8	1370	0.3	-	-	[31]

**Table 2:** Distribution of connector dimensions.

Lithium Disilicate		Zirconia	
Rectangular (a) × (b)	Trapezoidal (a) × (b) × 1 mm	Rectangular (a) × (b)	Trapezoidal (a) × (b) × 1 mm
5 × 3 mm	5 × 4 × 1 mm	5 × 3 mm	5 × 4 × 1 mm
5 × 2 mm	5 × 2 × 1 mm	5 × 2 mm	5 × 2 × 1 mm
4 × 3 mm	4 × 4 × 1 mm	4 × 3 mm	4 × 4 × 1 mm
4 × 2 mm	4 × 2 × 1 mm	4 × 2 mm	4 × 2 × 1 mm

**Table 3:** The connector volume and its dimensions.

Connector Type	Dimensions	Connector Volume (mm^3^)
Rectangular	5 × 3 mm	19.61
	5 × 2 mm	9.04
	4 × 3 mm	11.61
	4 × 2 mm	6.55
Trapezoidal	5 × 4 × 1 mm	10.98
	5 × 2 × 1 mm	3.98
	4 × 4 × 1 mm	9.10
	4 × 2 × 1 mm	3.35

## Data Availability

The data presented in this study are available upon request from the corresponding author.
